# Effects of a Wearable Assistive Device on Postural Control and Stability During Symmetric and Asymmetric Intermittent Trunk Flexion Tasks

**DOI:** 10.3390/bioengineering12050456

**Published:** 2025-04-25

**Authors:** Pranav Madhav Kuber, Ehsan Rashedi

**Affiliations:** Department of Industrial and Systems Engineering, Rochester Institute of Technology, 1 Lomb Memorial Dr, Rochester, NY 14623, USA; pmk2015@rit.edu

**Keywords:** biomechanics, balance, exoskeleton, motion analysis, awkward posture, trunk bending

## Abstract

Assistive devices, such as Exoskeletons (EXOs) can enhance endurance, but could inadvertently alter body mechanics, compromise balance, and elevate fall risk, particularly under fatigue. We evaluated effects of an EXO on postural stability during standing still and sustained trunk flexion tasks as users become fatigued during intermittently performed tasks. As trunk bending is common across many occupational/routine tasks, a repetitive 45° trunk flexion task was selected. In this controlled laboratory study, symmetric and asymmetric trunk flexion tasks were performed by twelve participants with a Back-support EXO until medium-high fatigue level (7/10 on Borg CR10 scale). Outcomes showed that the device increased trunk flexion durations (~16~25%), and upper-body movement beyond intended position. EXO-use improved stability by reducing maximum deviation (~22%) and mean velocity (~57%) of Center of Pressure (COP) co-ordinates. Asymmetric trunk flexion without assistance led to highest mean velocity of COP during fatigued state, but the same remained lower (~67%) with EXO-use, even with fatigue. The device decreased variance of COP during in medial/lateral direction (~44%), but increased the same in anterior/posterior direction by the same amount. Efforts in this study contribute towards understanding implications of using assistive devices for improving human performance across diverse applications.

## 1. Introduction

Postural control refers to the ability to maintain, attain, or regain balance, and depends on the coordinated integration of sensory inputs and motor responses to ensure stability and orientation throughout various activities. Imbalance is often a results of reduced psycho-physical and sensory capabilities of the individual [1,2]. Fatigue, often caused by performing repetitive demanding tasks, is one such factor that leads to reduced physical, cognitive, and/or muscular capabilities. This could not only cause task errors, but also increase injury risk [3]. Examining fatigue progression can help in developing interventions that minimize detrimental effects of fatigue [4,5,6,7]. For instance, fatigue-induced reductions in muscle force production are known to impact overall movement and postural stability [8,9]. For instance, fatigue affects dynamic stability in the spine and hip, contributing to improper control and increased fall risks [10]. Awkward postures, asymmetric loading, and manual material handling further impact sensory systems crucial for maintaining balance [11,12]. Evaluating the impact of fatigue on body movement and stability during tasks provides crucial insights into the risk of falls [13], contributing to the development of effective interventions.

Assistive devices, such as Exoskeletons (EXOs), are designed to augment human capabilities through assistive forces that may reduce overall physical demands and the progression of fatigue, potentially improving stability during prolonged tasks. EXOs are categorized based on the body region they support (e.g., back, shoulder and leg), structure (rigid, soft, hybrid), and actuation method (active/electromechanical, passive/pseudo-mechanical) [14]. Among the several types, passive ones are more likely to be adopted by users due to their affordability, compactness, and simplified structure. For instance, passive Back-support EXOs have been designed to alleviate low-back muscle effort by providing assistive torque to the upper torso using actuators located in the hip region [15]. Prior studies have investigated the efficacy of Back-support EXOs in reducing low-back muscle demands during bending [16,17,18,19] and lifting tasks [20,21,22,23,24,25,26], showing that Back-support EXOs can be helpful in alleviating low-back demands.

Although EXOs offer potential advantages in reducing muscle effort and fatigue progression, their usage may lead to adverse/side effects [27]. Such mixed effects of EXOs have also been found in studies conducted in real-world scenarios [14]. Such effects may be due to the additional weight of EXOs (ranging from 2.2 to 4.5 kg), and the application of assistive/resistive torques during activities can result in altered movements, reduced stability in terms of decreased deviation in the floor projection of center of mass, also termed as center of pressure (COP), diminished chances of recovery, and a potentially increased risk of falls [8,28]. In bending tasks, adopting a forward-bent posture may expose users to a risk of falling [29], particularly in cases where humans are required to assume awkward postures, such as asymmetric bending [26,30,31]. For example, a study by Park et al., 2021, showed that Back-support EXOs increased the COP median frequency and velocity during quiet bipedal standing, indicating reduced stability while wearing a Back-support EXO [8]. However, the external structure of a Back-support EXO can also enable a more controlled trunk motion, ultimately providing a more stable motion. Interestingly, contrary to findings from past studies, Back-support EXO was also found to be effective in improving stability. In a recent study [32], Back-support EXO decreased COP velocity by 23% during trunk flexion tasks. This could have been due to the increased weight, which may bring COP closer to the center of the body, reduce deviations in COP locations, as well as the body support provided by the structural components of Back-support EXO. Given the mixed outcomes of studies, concrete evidence on whether Back-support EXOs adversely affect stability is still needed. While it is often infeasible to conduct accurate and detailed real-world studies, controlled lab-based studies with simulated conditions can offer valuable insights.

Prior controlled laboratory-based studies have evaluated effects of EXOs on stability during quiet standing, static tasks, or tasks that were isolated [8,32,33,34]. However, most real-world can be distilled down to a combination of fundamental body movements (flexion/extension, rotation of body parts), and include both static and dynamic activities. Even in tandem with relaxation breaks, people experience fatigue when tasks are performed over prolonged periods. The novelty of our study lies in the unique intermittent task paradigm that was designed to examine effects of using an EXO on stability as their wearers become fatigued. Particularly, this study aims to answer the research question of what effects does a fatiguing task of performing trunk flexion tasks with an EXO cause on the spatio-temporal measures of trunk kinematics and whole-body stability.

### Study Objectives

Past studies have shown that affected neuro-musculoskeletal systems can lead to instability, especially in fatigued states [9,35,36]. Moreover, given the added weight and bulkiness of exoskeletons, any perturbations in movements can increase risk of instability, and the possibility to retain balance might be reduced [8,33]. With a flexed trunk posture and presence of fatigue in low-back and leg regions, EXO wearers could be prone to greater fall risk. Specifically, our research hypothesis was that using the Back-support EXO will lead to significantly lower values in trunk velocity and acceleration measures, but will increase instability as reflected in the velocity and deviation in measures of center of pressure (COP) location after examining a stabilogram. The broader part of this study involved studying impacts of using a Back-support EXO during intermittent trunk flexion tasks on overall physical demands including muscle activity and fatigue [37]. This study specifically focusses upon the effects of performing intermittent trunk flexion tasks with a Back-support EXO and its impact on trunk motion and whole-body stability.

## 2. Materials and Methods

### 2.1. Study Participants

Twelve young adults from 50th percentile stature male population were recruited from the university student community. The sample size of this study was decided based on the sample sizes used in prior similar lab-based studies (8–16 participants), where significant effects were detected in the measures of stability and trunk motion [8,20,38]. The required sample size, determined using a statistical effect-size approach, was also confirmed to be sufficient (minimum: 10) for the experimental design with a power of 0.8 and a Type-I error of 0.05 to detect “large” effect sizes (i.e., ω2 ≥ 0.15) [39]. Inclusion criteria specified participants within height: 152–182 cm, weight: 55–90 kg, engaging in exercise at least twice a week, and having no incidents of musculoskeletal disorders < 6 months prior to experimentation. For this controlled experiment, only males were recruited, as the device used for evaluation was reported to be uncomfortable by females during pilot testing. Since the study included perceived exertion ratings, which may be affected by such discomfort during use, we decided to exclude females for this specific study. We also measured stature, chest, and hip circumference, using a measuring tape and weight with a weighing scale prior to experimentation to ensure a proper fit of the Back-support EXO (Table 1). Written informed consent was obtained from all participants. This procedure adhered to the ethical standards set by the university’s institutional review board (HSRO#01113021) according to the Declaration of Helsinki.

### 2.2. Study Design and Apparatus

Trunk flexion is a fundamental movement commonly performed in daily life and across various occupations, yet it is also a well-documented contributor to low-back pain and musculoskeletal injury risk, particularly when sustained or repeated under load [35,40]. Real-world tasks rarely involve continuous, high-intensity activity without variation; instead, they typically consist of mixed-intensity movements interspersed with periods of rest or low-demand activity. Importantly, prolonged or repetitive trunk flexion can induce fatigue in the low-back and lower limbs, potentially compromising balance and motor control. To better reflect these conditions, we designed an intermittent task paradigm incorporating repeated cycles of trunk flexion, sustained flexion posture, quiet standing, and relaxed standing. This approach enables an assessment of fatigue-related changes in postural stability and motor performance, particularly in the context of wearable assistive device use.

As this was a controlled study, we decided to simplify real-world tasks, which are much more complex exhibiting asymmetry in multiple planes of torso rotation. The task was deliberately selected to align with the aims of this laboratory study. Although incorporating real-world tasks may enhance ecological validity, the inherent variability in uncontrolled body movements across trials could introduce inconsistencies and confound outcome measures. The chosen 45° trunk flexion task reflects a common movement observed in various occupational settings, including manufacturing (e.g., assembly or welding in a flexed posture), agriculture, and healthcare environments such as surgical procedures, thereby maintaining both experimental control and functional relevance.

**Figure 1:**
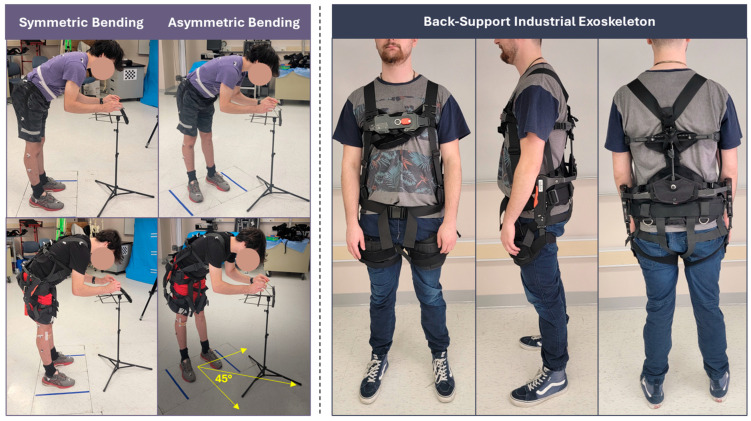
Schematic showing (**left**) the experimental setup, and (**right**) the SuitX Back-support exoskeleton.

The rotation angle of 45 degrees was selected based on our pilot studies, where higher angles could cause discomfort and lower values of angles did not produce sufficient observable differences. Our study undertook a task-centric approach of performing multiple task cycles until fatigue, where each task cycle comprised standing still, bending/retraction, and maintaining a bent posture in symmetric and asymmetric postures, performed intermittently with relaxation breaks. An asymmetric posture condition was selected [41], wherein the setup was placed ~45 degrees (towards left-side) in the transverse plane relative to the neutral position of the participant, as depicted in Figure 1. Symmetric postures were controlled by visually monitoring participant throughout the experimental session.

To evaluate temporal effects during task performance, participants were instructed to execute each task module (consisting of standing still, bending, sustaining a bent posture, retracting, and standing still) until participants reached a medium-high level of perceived fatigue in their low-back region. To investigate impacts of Back-support EXO, tasks were performed with/without the device. Thus, study conditions followed a 2 × 2 experimental design, with Assistance (No EXO/NE, Back-support EXO/E) and Posture (Symmetry/S, Asymmetry/A) as independent variables leading to the investigation of four conditions (S-E, S-NE, A-E, A-NE).

**Figure 2:**
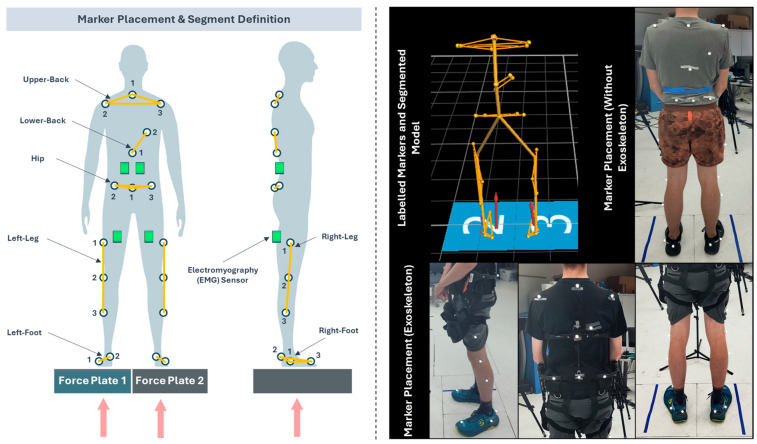
Schematic with (**left**) an illustration showing marker placement locations, and sensor systems, including force plates (under each foot) and electromyography sensors (green boxes), and (**right**) definition of segments for recording body movement using optoelectronic marker-based motion capture system. Red colored arrows indicate ground reaction forces.

To minimize motion-related artifacts in the data, we introduced a simplified forward bending task of grasping two wire connectors positioned approximately ~25 cm apart, as shown in Figure 2. The setup, including the wiring and stand, was adjustable for height and incline, with sternum angle ~45° in sagittal plane. We selected the SuitX BackX Model AC (US Bionics Inc., Emeryville, CA, USA) EXO consisting of a passive torque generation mechanism on each side of the wearer’s hip region. This specific device was selected due to its capability in supporting the torso during trunk flexion tasks. The device consisted of a combination of flexible straps and rigid metallic components, and weighed ~3.5 kgs. To maintain consistency and minimize potential interaction effects, we set the support level of the exoskeleton at a medium level, corresponding to ~25 pounds of support, which was determined as suitable during pilot studies where participants were able to perform sufficient number of trials (at least 10 trials per condition with the device). This support level was kept consistent across all participants throughout the study.

### 2.3. Data Aquisition

**Figure 3:**
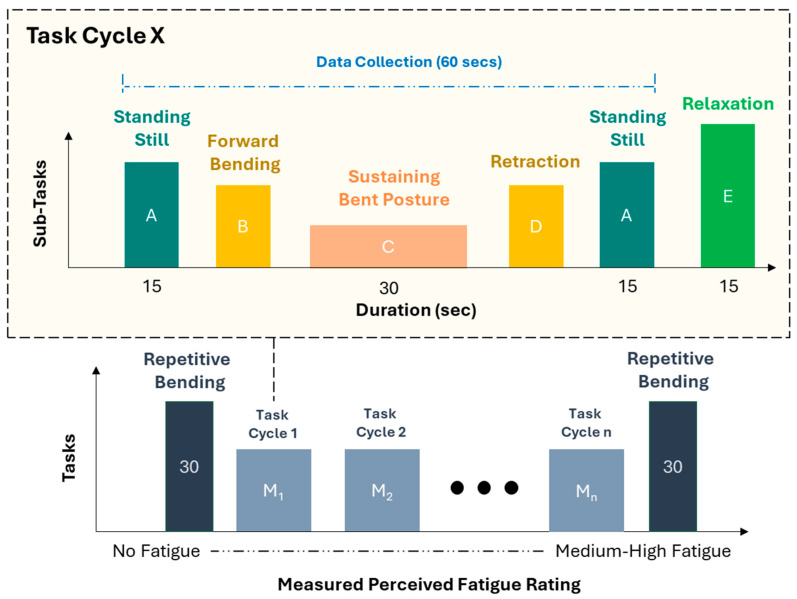
Schematic representing the experimental protocol with tasks and sub-tasks arranged in modules. Tasks included 30 counts of repetitive bending at the start during no fatigue, and then again at the end at medium-high fatigue with sub-tasks in the form of task cycles in between. Sub-tasks were conducted for a total duration of 75 s including a 15 s relaxation break.

We used an optoelectronic motion capture system (VICON, Hauppauge, NY, USA) to record body movement at 100 Hz with placement of 20 markers on the torso (3 on the upper back, 2 on the middle back, 3 on the hips) and lower body (3 on each leg and foot). Marker placement locations on the lower-body and back side of trunk were determined based on guidelines in the VICON Nexus Documentation for the Plug-in Gait Model [42], and defined in Figure 3 with segments for upper-back, lower-back, hip, left/right leg, and left/right foot. Markers were placed on the back and side regions of the body to ensure visibility to the camera system. To prevent detachment during experimentation, additional tapes were affixed to all sensors and markers. Surface Electromyography (sEMG) sensors were also placed on the low-back and the back of upper-legs (thigh) regions and analyzed, but were not included in this article due to the scope of this study. The outcomes of muscle activity and fatigue assessment can be found in our recent publication [43]. Each sEMG sensor included an Inertial Measurement Unit (IMU) sensor, which was utilized for identifying start/end portions of tasks.

Whole-body stability was mainly assessed during static standing at start/end and sustained bending portions of each task cycle. An irregular/wobbly motion in the human body could be caused due to hindrance in the ability to maintain balance. For evaluating these effects on human stability, movement variations associated with the Center of Pressure (COP) can be assessed using force plates [11,44]. Prior studies have assessed the effects of conditions/interventions, particularly neuromusculoskeletal disorders and effects of wearable assistive systems [44,45,46]. In this study, we used a force plate system, comprising two separate force plates (AMTI, Advanced Mechanical Technology, Watertown, MA, USA) with sampling frequency of 1000 Hz, and participants stood with one foot upon each of the two floor-embedded force plates throughout the experiment.

Lastly, fatigue was assessed using perceived ratings of exertion using Borg CR10 scale [47]. This scale, ranging from 0 to 10, provided a nuanced representation of perceived fatigue, with descriptors of ‘no exertion—0’, ‘very slight—1/2’, ‘slight—3’, ‘moderate—4’, ‘somewhat severe—5’, ‘severe—7’, ‘very severe—9’, and ‘maximal—10’.

### 2.4. Experimental Tasks and Procedure

The study comprised three sessions (training/session-1, session-2, session-3), each conducted on separate days with >48 h between sessions. The primary objective of the initial session (~1 h) was to provide training and acquaint participants with the equipment, tools, experimental tasks, and overall protocols. After obtaining consent, participants engaged in a wall-sit task, vocalizing their perceived exertion ratings until reaching maximal fatigue on a subjective (Borg CR10) scale for self-calibrating their perceived fatigue levels [48]. The initial session concluded with participants performing tasks in all four conditions and familiarizing themselves with the Back-support EXO through walking and bending activities.

Following the training session, each of the two subsequent sessions involved participants performing tasks either with or without assistance in both asymmetric and symmetric postures, counterbalanced as well as randomized in order. After completing all trials for the first condition, participants were given a rest break until their fatigue level reached very slight/slight fatigue (1/2) in the back region on the Borg CR10 scale. Each session under the no exoskeleton (NE) condition lasted approximately 2–3 h, encompassing both Symmetry (S) and Asymmetry (A) conditions. Conversely, sessions with the exoskeleton (E) condition took about 4–5 h. Variation in the duration of sessions across participants was observed. This was primarily due to the capacity of performing tasks being much higher. We also ensured that discomfort experienced by participants was minimal, and participants were provided short breaks of 15 s due to the intermittent nature of the tasks, which also relieved any potential discomfort caused due to the static nature of posture maintenance tasks. Rest breaks between the Symmetry and Asymmetry conditions within the same session lasted approximately 15–25 min. Figure 3 illustrates the protocol for each condition, involving 30 cycles of repetitive bending at the start (RPE in back: 0 (no exertion)) and at the end (RPE in back: 7 (very severe)). Intermittent bending task modules were inserted between these cycles. Each task module included standing in a neutral posture for 15 s, sustaining a bent posture while grasping wire connectors for 30 s (holding two separate wires together), standing in a neutral stance for 15 s, and relaxing for 15 s. These durations were determined based on the time needed for increments in fatigue levels and completing each session. Participants were allowed to move their torso and limbs only during the relaxation period to alleviate discomfort, and they were instructed to avoid any body movement while performing other tasks. Participants were asked about their perceived fatigue level in back and leg regions after performing each task cycle for all experimental trials, which was noted down along with the task cycle number.

While data were collected representing muscle activity and demands, upper and lower-body motion, and stability, only trunk motion and stability data were utilized for this study. Data collection included repetitive bending at start/end, as well as for each task module (60 s period × number of completed trials). After completing all of the tasks for the session, participants were asked to rest for 10 min. Once two conditions (symmetric and asymmetric bending, with/without Back-support EXO) were completed, the session was concluded. The experiment was concluded once participants completed two sessions consisting of performing all study.

### 2.5. Data Analysis

**Figure 4:**
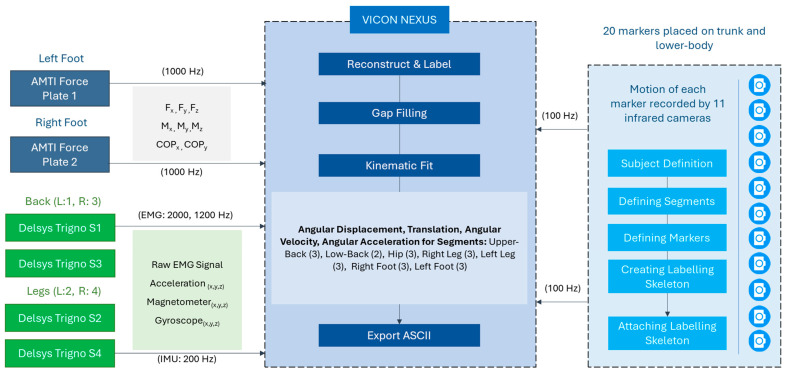
Schematic depicting an overview of the VICON system showing time-synced data collection from surface electromyography (sEMG), motion capture, and force plate systems (all data except those collected from sEMG were utilized for analysis in this study).

Time-synced data were obtained using the VICON lock studio and lock lab hardware and Nexus 2.0 software, which enabled exporting of data from all three systems in the form of a single (.csv) Excel file, as depicted in Figure 4. The collected data were imported into MATLAB (R2021b) and processed using a custom-developed code to obtain insights on changes in body movement and whole-body stability with conditions. Activities were segmented based on the data obtained from IMU within sEMG sensors to separate standing still, bending, retraction, and sustained bending activities for each task cycle. The resultant of raw acceleration obtained from the IMU sensor in the right low-back region was used to identify task activities and segment data into portions representing each of the task activities. The developed code would detect the start/end instances of motion, and segment tasks accordingly based on pre-defined percent thresholds of the base signal. As per the scope of this article, all data except that for sEMG was analyzed in this study. Start/end portions of each activity were excluded (e.g., first 5 s of sustaining bent posture) to account for data fluctuations. Measures from the markers were extracted relating to movement of upper-body during each of these activities within task cycles by considering the upper-back marker. The angles of trunk flexion in sagittal, coronal, and transverse planes were calculated using the upper-back and hip markers. For the ‘with Back-support EXO’ condition, correction was applied to create a virtual marker for comparing trunk angles between conditions. The exoskeleton included rigid links that protruded outwards from the low-back skin. Thus, we could not place the actual reflective marker during with exoskeleton condition in the same placement location as the placement location without exoskeleton condition. Thus, we considered a correction factor that represented the additional distance between skin and the exoskeleton structure to create a virtual marker, whose co-ordinates were utilized for calculating kinematic measures. Subsequently, velocity and acceleration of upper-back and hip markers were calculated and compared using a range of parameters (Table 2). This included the durations of dynamic movements (bending/retraction), as well as velocity measures during sustained bending task. In addition, we calculated percent overshoot to quantify the additional movement to reach stable neutral posture that occurred after completing the entire range of motion of dynamic activities. As observed during pilot studies, presence of fatigue and additional weight of the exoskeleton may lead to more unintentional motion and longer time to reach a stable posture. Meanwhile, for stability analysis, co-ordinates of the Center of Pressure (COP) obtained from force plates were considered similar to prior studies [2,8,49]. The stabilogram is a representation of the movement of the COP location over time on a flat plane. Higher deviations in the stabilogram are an indicator of affected motor control capabilities and comparison of values across conditions can be insightful in understanding instances of more instability. Mean velocity represented the intensity of body movements that occurred while the participant tried to maintain a stable posture. With an affected system, either due to fatigue, asymmetric posture, or wearing the exoskeleton, motor control ability could deteriorate, leading to more movements over the period. Similarly, maximum deviation was an indicator of the maximum extent of the shift of the COP location in either direction of the horizontal plane. Both parameters of mean velocity and maximum deviation were calculated by differentiating the location co-ordinates followed by an average value of the resulting velocity datapoints, and by determining the peak value in the COP location signal in both directions of the horizontal plane. Besides these two parameters, we also calculated sample entropy and variance in the COP location signal. Lastly, differences in ground reaction forces (GRF) at the left vs. right foot as well as their mean values were determined for each condition by calculating the resultant GRF at each foot from the obtained raw data.

The final set of measures, shown in Table 2, pertain to the analysis of movement and balance during a task involving bending and retraction phases. For instance, trunk flexion durations represent the time in seconds taken to complete the bending and retraction movements, respectively. The maximum upper-back velocities were the peak velocities of the upper-back marker 1 (UB1) in three spatial directions—x (anterior/posterior), y (medial/lateral), and z (superior/inferior)—during the sustained phase. Overshoot percentages quantify the extent of motion beyond expected displacement during or after bending/retraction, suggesting possible instability or momentum effects. Spatial coordination is further examined via distance between COP and upper-back locations, which indicates the horizontal distances between upper-back marker and the center of pressure (COP) co-ordinates during sustained bending posture. COP stability metrics included maximum deviation and mean velocity across different task phases—standing at the start (SS), sustained bending (SUS), and standing at the end (SE)—offering insights into postural sway and control. Advanced dynamics are captured through Sample Entropy and variance in COP, representing the complexity and variability of COP trajectories during sustained portion of the task. Sample entropy is a nonlinear metric used to quantify the complexity or regularity of the movement of the center of pressure (COP) [50]. Lower values indicate more regular or predictable patterns of COP movement, which may reflect rigid or constrained control strategies, while higher values suggest greater irregularity or complexity, reflecting a more adaptive and flexible postural control system that responds dynamically to small perturbations. Ground reaction force asymmetries and load distributions were assessed with difference in GRF between left and right foot and their mean values, highlighting how force is shared between the lower limbs. Collectively, these measures provide a comprehensive view of movement kinematics, postural control, and load distribution during functional bending and retraction tasks.

A (2 × 2) model was created using the factors: Assistance (AST): with exoskeleton (E)/without exoskeleton (NE) and posture (P): with asymmetry towards left (ASY)/without asymmetry (S), with personality set to Standard Least Squares with an emphasis on Effect Leverage. Participant numbers were first nested within posture factor and then randomized to account for within participant variations due to primary interest in understanding effects of assistance. Calculated measures were reported in the form of mean (SD) and statistical comparisons were made to assess significant differences. This was followed by post hoc paired comparisons using Tukey’s Honest Significant Difference (HSD) Test where relevant. All statistical analyses were conducted using JMP Pro 14^®^ (SAS Institute, Cary, NC, USA), with the statistical significance level of *p*-value < 0.05. Parametric model assumptions were verified before considering the results. Outcomes were reported as mean (SD) of the values for each trial classified according to low (0–3) and medium (3–6) fatigue, based on RPE ratings provided by the participants.

## 3. Results

A statistical summary of significantly different comparisons including *p*-values have been depicted in the Appendix A for both trunk movement and stability measures. We also categorized the measures based on RPE scores from 0 up to 3 as “low” and scores from 4 to 6 as “medium”. Following sections describe the effects on movement and stability.

### 3.1. Impact on Body Movement

#### 3.1.1. Duration of Bending and Retraction Movement

**Figure 5:**
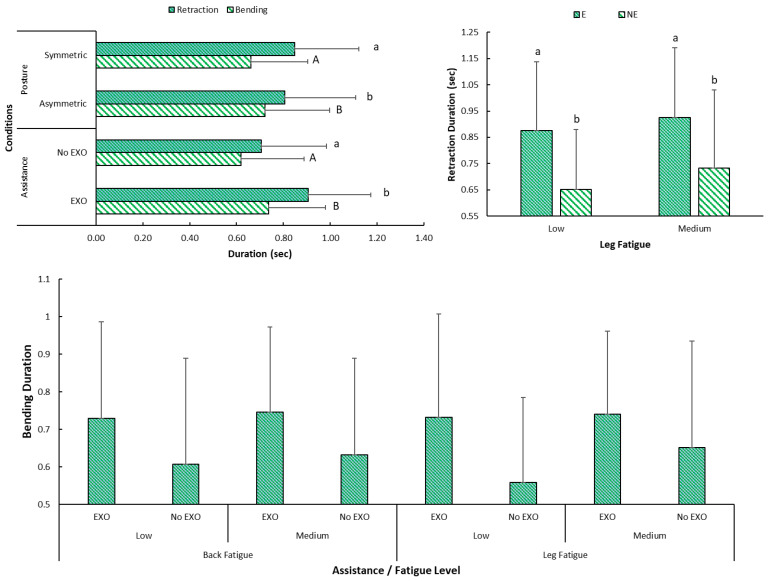
Graphs showing (**left**) duration of bending and retraction categorized according to assistance (No EXO: no exoskeleton, EXO: exoskeleton) and posture (asymmetric/symmetric), and (**right**) retraction duration variation with leg fatigue. Bottom graph also includes categorization based on back and leg fatigue as low fatigue and medium fatigue (note: Significant differences are denoted by different letters in different style in the graphs at the top. Values between conditions within same sub-category are significantly different in the bottom graph. Error bars denote standard deviation).

The time taken for performing bending motion was higher when performing asymmetric tasks (A: 0.72 (0.27) s, S: 0.66 (0.24) s), and was also higher when wearing the Back-support EXO (E: 0.73 (0.24) s, NE: 0.62 (0.26) s) with *p*-value < 0.01. However, retraction took slightly less time in asymmetric vs. symmetric bending (Figure 5). Retraction durations were higher with Back-support EXO (E: 0.90 (0.26) s, NE: 0.70 (0.27) s) with *p*-value < 0.01.

#### 3.1.2. Trunk Velocity and Acceleration

**Figure 6:**
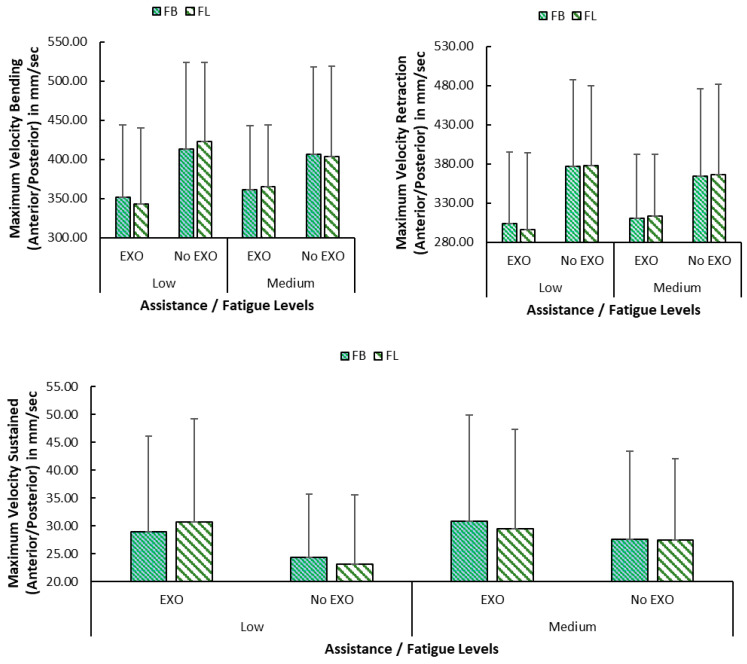
Graphs showing (**left**) maximum velocities in anterior/posterior direction during bending, retraction, and sustained bending portions of the task cycle categorized according to assistance (No EXO: no exoskeleton, EXO: exoskeleton) based on back (FB) and leg (FL) fatigue as low fatigue and medium fatigue (note: Values between assistance conditions within same category are significant in all graphs. Error bars denote standard deviation).

The bending angle in sagittal plane during sustained portion of task cycle was similar in all conditions except for asymmetric posture while wearing the exoskeleton wherein participants performed bending with ~5 degree less (Table 3). During sustained portion, maximum velocity in sagittal plane of the upper back was higher at 29.4 (18.07) mm/s with Back-support EXO vs. without at 26.06 (13.97) mm/s (*p*-value = 0.001) (Figure 6). The same in sideways direction was slightly higher with more variation at 21.7 (14.95) with Back-support EXO vs. 20.2 (7.7) mm/s (*p*-value = 0.01). Lower values of maximum velocity and less variation were seen during bending portion of the task cycle in all three directions (Table 4).

The maximum angular acceleration of the trunk during bending in the sagittal plane was higher without Back-support EXO assistance (EXO: 28.8 mm/s^2^, No EXO: 33.6, *p* < 0.001) (Table 5). Similar was the case during retraction where higher angular acceleration of the trunk was seen (EXO:32.1, No EXO: 36.6). Slightly lower values (~2%) were seen for asymmetric postures and values increased by <3% when fatigue was medium vs. low.

#### 3.1.3. Percent Overshoot During Movement

Table 6 shows the variation categorized according to posture and assistance. Use of Back-support EXO led to higher percent overshoot at the end of retraction in the front/back direction of 6.73 (4.30) % as compared to without using the device (5.86 (3.6)) (*p*-value < 0.001). Specifically, the highest values were seen in asymmetric posture with Back-support EXO, while lowest values occurred without Back-support EXO for symmetric postures.

#### 3.1.4. Relationship Between Sustained Posture and Stability

The relationship between posture during sustained bending and stability was assessed by examining distance between the COP in x (front/back) and y (sideways) directions and the marker across varying conditions. The distance between COP location and upper-back marker in the anterior/posterior direction was higher without the Back-support EXO at 183.55 (35.5) mm vs. with assistance at 173.89 (47.76) mm (*p*-value < 0.001). However, in the medial-lateral direction, distance was higher with the assistance at 91.31 (68.5) mm vs. 84.15 (65.1) mm (*p*-value < 0.001). Based on posture, distance in the anterior/posterior direction in the asymmetric posture was (150.4 (40.7)) mm, and was lower than in symmetric posture (203.3 (27.7)) mm (*p*-value < 0.001). This value was higher in both symmetric (by ~10 mm) and asymmetric (~15 mm). When leg fatigue was medium (*p*-value = 0.018), a higher distance of 183.7 (41.3) mm was seen vs. during low leg fatigue in anterior/posterior direction (167.12 (45.36)). In the medial/lateral direction, higher values were seen for low vs. medium leg fatigue, and distance between COP and back marker was highest when wearing Back-support EXO at low leg fatigue at 93.18 (64.9), and was the lowest without assistance in medium leg fatigue (83.42 (64.7)) mm (*p*-value < 0.001).

### 3.2. Impact on Stability

Several measures of stability showed significant differences based on use of assistance, and posture conditions as depicted in Table 7 and Table 8. Higher values were observed when Back-support EXO was not worn in measures of maximum deviation, mean velocity, sample entropy (in both directions), variance (sideways) of COP, and difference in left/right ground reaction forces. When categorized based on posture, higher values were observed for asymmetric posture in the measures of maximum deviation, mean velocity, variance (in sideways direction) of COP location, and difference between left/right forces. Specifically, both left and right ground reaction forces were higher when Back-support EXO was worn. As expected, ground reaction forces were higher in the left force plate and were lower in right force plate during asymmetric bending towards the left vs. right side.

#### Effects of Asymmetry and Fatigue on Stability

**Figure 7:**
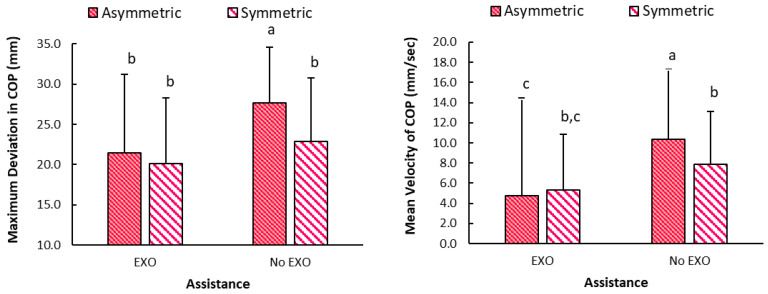
Graph showing maximum deviation and mean velocity of COP location during sustained portion of task cycle categorized based on assistance (NE: no exoskeleton, E: exoskeleton), and posture (A: Asymmetric, S: Symmetric) (note: Different letters (a/b/c on error bars denote statistical significance. Error bars denote standard deviation).

**Figure 8:**
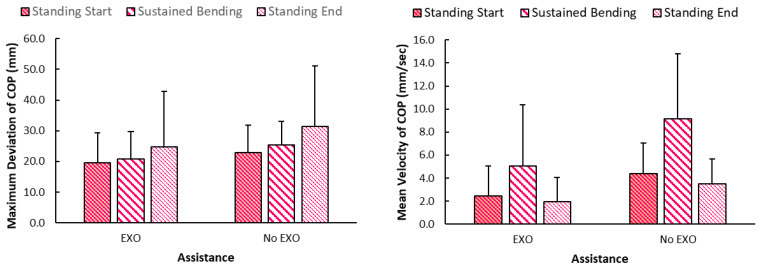
Graph showing maximum deviation and mean velocity of COP location during standing still at the start, sustained and standing still at the end portion of task cycle categorized based on assistance (No EXO: no exoskeleton, EXO: exoskeleton), and posture (Asymmetric, Symmetric) (note: Values between assistance conditions (E vs. NE) are significantly different for each portion of task cycle. Error bars denote standard deviation).

**Figure 9:**
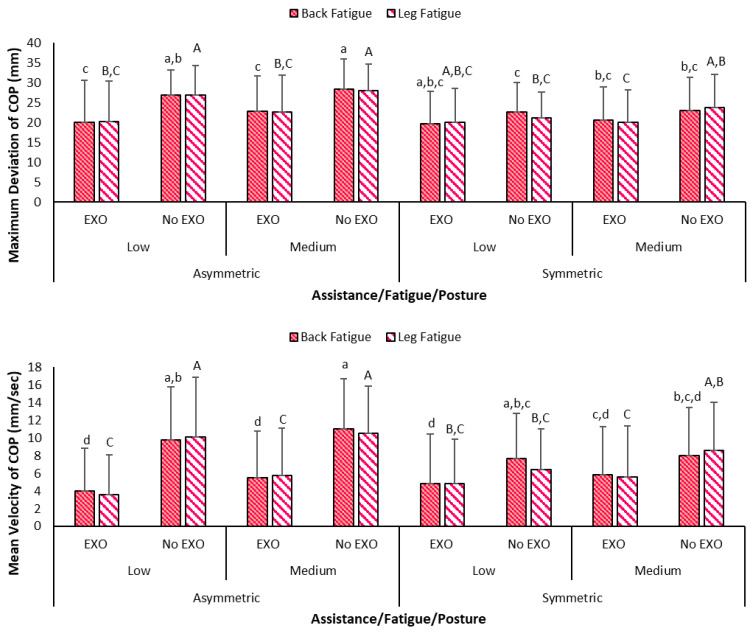
Graph showing maximum deviation and mean velocity of COP location during sustained portion of task cycle categorized based on assistance (No EXO: no exoskeleton, EXO: exoskeleton), back/leg fatigue (Low, Medium) and posture (Asymmetric, Symmetric) (note: different styled letters on error bars denote statistical significance. Error bars denote standard deviation).

The comparison between asymmetric and symmetric postures vs. assistance has been shown in Figure 7, wherein highest values were seen for without assistance in asymmetric postures. Highest values for both maximum deviation and mean velocity of COP were seen for asymmetric postures during medium back fatigue levels. Specifically, maximum deviation of 25.22 (8.67) mm was the highest maximum deviation vs. the lowest of 20.7 (7.9) mm during symmetric posture at low fatigue. Similarly, mean velocity of 7.73 mm/s was seen in asymmetric/medium back fatigue vs. lowest of 5.8 (5.5) mm/s during symmetric at low back fatigue. Meanwhile, Figure 8 shows the variation in maximum deviation and mean velocity measures between standing at start/end and sustained bending portions. Maximum deviation of COP was the highest during standing at the end, and values were higher without Back-support EXO. On the other hand, mean velocity values were highest during sustained portion as compared to standing still. As seen in Figure 9, similar values were seen when categorized according to back vs. leg fatigue levels. Higher values were seen in all conditions when Back-support EXO was not used. Specifically, highest values were observed for the asymmetric conditions without the use of assistance.

Significant differences were seen in the variances in sideways (medial-lateral) direction across back and leg fatigue when categorized based on posture and assistance conditions. The values were higher in asymmetric postures between without vs. with Back-support EXO conditions, as shown in Table 9. Specifically, asymmetric postures led to ~20–30 mm^2^ higher values without wearing the exoskeleton vs. when wearing the device.

## 4. Discussion

This study incorporated an intermittent task cycle paradigm with static standing, bending/retraction, and sustained bending tasks, performed until medium-high fatigue levels (RPE:7) in low-back region with/without Back-support EXO and with/without asymmetry. This study aimed to understand the effects of wearing a back assist device, Back-support EXO, on trunk movement and whole-body stability of wearers. A key strength of this study lies in its use of an intermittent task paradigm, which represents a novel approach compared to the isolated, single-phase tasks commonly employed in previous research. While past studies often focused solely on static bending or discrete movement [51,52,53], the intermittent design integrates repeated cycles of static, sustained postures, and dynamic actions—more closely mimicking real-world activities that include transitions and prolonged engagement, yet in a controlled lab-based study. This paradigm enables the capture of temporal patterns, fatigue-related adaptations, and recovery behaviors that isolated tasks may overlook. Additionally, it allows for the assessment of how motor control strategies evolve across task repetitions, providing richer insight into movement efficiency, postural stability, and compensatory mechanisms. This continuous, multi-phase approach offers a more ecologically valid and functionally relevant framework for evaluating postural control and stability.

### 4.1. Implications for Trunk Kinematics

Prior studies have assessed effects of using an EXO on kinematics, yet the measures utilized mostly included variations in joint angles. For instance, increases in median knee (6%) and hip flexion angles (11%) when using an EXO were reported in one study [54], and deviations up to 14° in knee, hip and lumbar joint angles during work postures with EXO in another [28]. This study used more in-depth measures for kinematic analysis. We found that bending/retraction movements were slower with Back-support EXO, causing ~16% increased bending and ~25% retraction durations. Agreement was also seen in measures of velocity in sagittal plane (~20–25% lower with Back-support EXO) of the upper-back marker, as well as the angular velocity of trunk segment (upper-back and hip center markers). Maximum angular acceleration of trunk during bending and retraction in the sagittal plane was ~11 and ~15% lower with Back-support EXO, respectively. This slower movement may have been caused by either the additional weight of the device, or the restrictions in natural movement due to several attachments of the device. Overall, participants took ~6% more time when bending in asymmetric postures, but took 4% less time retracting back to neutral posture than symmetric postures. Higher duration may have originated from the single muscle being used to stop the movement. Meanwhile, symmetric postures required complete trunk flexion and participants covered more distance vs. asymmetric postures. Participants twisted their mid-torso rather than hips, which made coming back to neutral posture faster. We also observed that back fatigue influenced bending/retraction durations (Figure 5). Performing the tasks during medium back fatigue with Back-support EXO had highest retraction time of 0.93 (0.2) seconds (*p* < 0.05), which was expected as more muscle force was required to bring the already higher weight (upper body plus Back-support EXO) to neutral posture.

Another unique measure used in this study included the percentage overshoots. The same of upper-back in downwards direction was highest during start (~8% of complete range of motion) and end (~3%) of bending without Back-support EXO in symmetric postures, and may have been the result of higher velocity. However, percent overshoot in anterior (backwards) direction at the end of retraction was the highest (~8%) in asymmetric postures with Back-support EXO (*p* < 0.01). Thus, higher overshooting may have been the result of reduced capabilities in postural control and differences in muscle co-activation due to the nature of the task. Although Back-support EXO led to ~20–25% lower bending and retraction maximum velocity of upper-back in anterior/posterior direction, the maximum velocity with Back-support EXO was ~10% higher during sustained portion. The lowest value was seen without Back-support EXO at low fatigue levels, which significantly increased for medium fatigue level categorization. This could mean that the higher maximum velocity during sustained bending posture may have been the result of discomfort in the form of a reaction towards relieving strain caused by maintaining a fixed posture.

### 4.2. Relation Between Trunk Motion and Stability

The relation between body movement parameters and whole-body stability is often examined together. For instance, kinematic parameters such as hip flexion, and knee range of motion were measured to explain movement impedance and lack of recovery from slips when wearing an EXO [33]. In this study, we determined the relative distances between the upper-back and the COP location. The distance between COP and upper-back central marker in the anterior/posterior direction was higher without the Back-support EXO at 183.55 (35.5) mm vs. with assistance at 173.89 (47.76) mm (*p*-value < 0.01). This difference may have originated from the location of hips during bending. For instance, without Back-support EXO assistance, participants often moved their hips backwards to have a more stable posture, wherein COP location during bending would lie further backwards. While wearing Back-support EXO, the structural components and the support in bending postures possibly prevented wearers in moving their hip backwards. In the medial–lateral direction, the overall distance was ~8% higher with Back-support EXO. This value was higher in both symmetric (by ~10 mm) and asymmetric (~15 mm) postures when Back-support EXO was worn.

### 4.3. Effects on Stability and Potential Impacts on Fall-Risk

Effects on balance have been studied quantitatively by examining whole-body stability [50] and postural sway [55]. A recent study also evaluated standing balance of two exoskeletons (Laevo, SuitX) where findings showed that wearing the EXO increased mean velocity of the COP series in the anterior–posterior direction by 7% [8]. In contrast, findings of the stability analysis in this study demonstrated that Back-support EXO led to more stable sustained bending postures with reduced maximum deviation by ~22% and mean velocity by ~57%. This contrasted with our earlier expectations that EXO is reported to lead to adverse effects on balance during flexed trunk postures. These findings also align with one study where body sway reduced by 40 to 70% of total sway area when an EXO was worn during standing [38]. Meanwhile, asymmetry induced higher values in maximum deviation by approximately 13%, mean velocity by around 17%, variance (medial/lateral) of COP location by roughly 40%, and the difference between left and right forces by 29 N (Figure 9). Throughout this task, lower values of variance (medial/lateral) of COP by approximately 44% were observed with Back-support EXO. However, the same measure was higher by about 43% in the anterior/posterior direction. Back-support EXO constrained sideways body movement, redirecting it into more front/back movement during sustained bending activities. The added weight of the device may have contributed to the better values of stability measures during static and sustained postures. A past study showed that range of medio-lateral movement and sway area changes with additional loads were lesser when wearing an EXO vs. without the device.

### 4.4. Study Limitations

The sample size in this study was relatively small, consisting of only 12 participants, all adult males representing the 50th percentile stature category. As such, the findings may not fully generalize to broader populations, particularly females, who may exhibit different biomechanical responses, postural control strategies, and fatigue profiles due to anatomical and physiological differences. Future studies should include more diverse participant groups across sex, body sizes, and age ranges to better understand the variability in responses to wearable assistive devices and ensure inclusive, user-centered design. In the future, experiment in this study can be repeated with a larger and more inclusive sample size to obtain more generalizable outcomes. While this study focused only on the trunk kinematics, and whole-body stability, studying lower-body kinematics, especially during dynamic asymmetric activities, can offer further insights. Lastly, there was a natural a tendency in participants to lean toward one side (preferring either left or right muscle activation) when performing the tasks, which might have influenced postural sway and stability measures. Lastly, a limitation of this study is the use of a controlled laboratory task rather than a real-world occupational activity. While this approach allowed for consistency across trials and minimized variability in body movements, it may not fully capture the complexities and dynamic demands of real-world tasks. As such, the findings may have limited generalizability to occupational settings where trunk flexion occurs under more variable and unpredictable conditions, such as in manufacturing, agriculture, or healthcare environments.

The exoskeleton model used in this study was selected based on its commercial availability, ease of use, and relevance to industrial applications involving trunk flexion. It provided passive mechanical support to the lower back using hip actuators, aiming to reduce lumbar loading during bending tasks. A moderate support level was chosen to reflect realistic usage conditions where assistance is sufficient to alleviate physical effort without overly constraining natural movement. However, this choice presents certain limitations. The findings may not generalize to other exoskeleton models with different mechanical designs, active control features, or varying degrees of support. Additionally, the study evaluated only one fixed support setting, which may not capture how users respond to adjustable or task-specific support levels. Future research should explore a broader range of exoskeleton configurations and support intensities to better understand their impact on movement dynamics, postural control, and user adaptation across diverse populations and task demands.

### 4.5. Future Scope

Future assessments of postural stability can be enhanced by incorporating additional measures such as base of support (BOS) characteristics and responses to external perturbations, as seen in one recent study [33]. Analyzing BOS, defined by the area within the perimeter of contact between the body and the supporting surface, can provide insight into how individuals adjust their stance to maintain balance under different task demands. Narrow or asymmetrical BOS configurations, for example, may indicate compromised stability or compensatory strategies. Similarly, evaluating responses to controlled perturbations (e.g., sudden platform shifts or force impulses) can reveal the robustness of reactive postural control mechanisms [53]. Another method utilized by one study included examining the limits of stability, where maximum extent of body lean towards the side was measured [38]. These perturbation-based assessments offer a dynamic and challenging context to examine how quickly and effectively the body can detect and correct balance disturbances. Such measures could complement existing metrics to offer a more comprehensive understanding of postural control.

The intermittent task paradigm employed in this study, which involves repeated cycles of bending, sustained posture performed until fatigue, can be applied to scenarios well-beyond evaluation of EXOs. For instance, the designed model provides a clinically relevant framework for assessing functional movement, postural control, and fatigue-related adaptations. This model can be particularly valuable in diagnosing and monitoring musculoskeletal or neuromotor disorders, such as low back pain, or age-related postural instability. By analyzing detailed kinematic and kinetic measures—such as duration of movement phases, velocity profiles, center of pressure (COP) dynamics, and asymmetries in ground reaction forces—clinicians can identify aberrant movement patterns, instability, or compensatory mechanisms that may not be evident through static or single-phase assessments. Furthermore, the model’s sensitivity to subtle changes in performance over time makes it suitable for evaluating the effectiveness of rehabilitation interventions, ergonomic adjustments, or assistive devices. In occupational or geriatric health, this approach can help in early detection of fall risk or fatigue-induced motor decline, guiding personalized therapy plans aimed at restoring safe and efficient movement. In sports science, this model can be helpful in the analysis of postural control and force distribution under intermittent loading, can support targeted training and return-to-play decisions by ensuring athletes regain proper movement control before resuming training. Overall, this paradigm could be a valuable tool across diverse domains.

## 5. Conclusions

This controlled lab-based study evaluated effects of using an assistive device, a Back-support EXO, in intermittent bending tasks (including static standing, bending/retraction, and sustaining a bent posture), performed with/without ~45° asymmetry. Motion analysis findings revealed that the device EXO increased bending and retraction durations, but reduced angular acceleration of the trunk during bending and retraction. However, the Back-support EXO caused a higher percent overshoot at the end of retraction in the backward direction, notably pronounced in asymmetric postures. During sustained bending, the device affected natural body postures, particularly in asymmetric postures, where distinct differences were observed in the sagittal and coronal planes. Back-support EXO reduced upper-back maximum velocity in the anterior/posterior direction during bending and retraction, but was higher during sustained bending portion.

The stability analysis demonstrated that Back-support EXO led to more stable sustained bending postures, evidenced by a reduction in both extend and velocity of movement of center of pressure. In asymmetric postures, Back-support EXO proved beneficial by reducing the difference between ground reaction forces at the left and right foot. Notably, although slightly higher values were observed with fatigue, the presence of medium-high fatigue did not significantly impact whole-body stability.

Overall, the assistive device led to:

- Slower dynamic movement.
- Improvement in overall stability during static and sustained bending.
- Reduced maximum deviation by ~22% and mean velocity by ~57% during sustained bending.
- Reduction in variance in center of pressure by ~44% in medial/lateral direction during sustained bending.
- Higher variance in center of pressure by ~43% in anterior/posterior direction during sustained bending.

While the controlled nature of this study may limit direct generalization to real-world settings, the findings provide important foundational insights into trunk flexion mechanics and postural stability, offering a basis for future studies in field environments.

## Figures and Tables

**Table 1:** Demographic measurements of the study participants showing the mean, standard deviation (SD), and the ranges in the form of maximum and minimum values for age, height, weight, body-mass index, and chest and hip circumferences.

Factor	Mean	SD	Maximum	Minimum
Age (yrs.)	19.41	1.38	22	18
Height (cm)	178.33	3.34	184	172
Weight (kg)	73.66	6.36	82.44	65.68
Body-Mass Index (kg/m^2^)	23.16	2.28	27.56	20.50
Chest Circumference (mm)	89.83	4.09	96	84
Hip Circumference (mm)	86.91	6.11	98	80

**Table 2:** Response variables of trunk movement and stability. Tasks include B: bending, R: retraction, SUS: sustained bending, SS: standing at start, SE: standing at end of task cycle.

Response Variable (Unit)	Task	Description
Bending duration (s)	B	Duration of trunk flexion movement in seconds.
Retraction duration (s)	R	Duration of return to neutral stance movement in seconds.
Maximum velocity of upper-back (mm/s)	B, R	Maximum velocity of upper-back in anterior/posterior, medial/lateral, and superior/inferior directions.
Percent overshoot of upper-back during bending (%)	B	Movement of upper-back beyond intended position before starting/after ending the bending movement in superior direction in percentage.
Percent overshoot of upper-back (%)	R	Movement of upper-back beyond intended position after completing the retraction movement in anterior/posterior direction in percentage.
Distance between upper-back and COP (mm)	SUS	Distance between the upper-back and the center of pressure anterior/posterior, medial/lateral, and superior/inferior directions.
Maximum deviation of COP (mm)	SUS, SS, SE	Maximum deviation of the center of pressure co-odinates in the horizontal plane.
Mean velocity of COP (mm/s)	SUS, SS, SE	Mean velocity of the center of pressure co-odinates in the horizontal plane
Sample Entropy of COP (units)	SUS	Sample entropy of the center of pressure in anterior/posterior, and medial/lateral directions.
Variance in COP (mm^2^)	SUS	Variance of the center of pressure in anterior/posterior, medial/lateral, and superior/inferior directions.
Difference in Ground Reaction Force (N)	SUS	Difference between the ground reaction force at left and right foot.
Mean Ground Reaction Force (N)	SUS	Mean of ground reaction forces at the left/right force plate.

**Table 3:** Mean and standard deviation of trunk angles in sagittal, coronal, and transverse planes categorized according to assistance and posture (note: statistically significantly different values are shown by different letters in the ‘Diff’ column).

Assistance	Posture	Sagittal Plane Angle (deg)			Coronal Plane Angle (deg)			Transverse Plane Angle (deg)		
		Mean	SD	Diff	Mean	SD	Diff	Mean	SD	Diff
EXO	Asymmetric	48.9	8.4	A	35.8	9.3	A	39.7	9.6	A
	Symmetric	54.4	6.2	B	5.6	4.7	B	15.9	5.3	B
No EXO	Asymmetric	54.1	6.2	B	52.7	9.9	A	38.7	10.3	A
	Symmetric	56.1	6.2	B	8.5	8.1	B	12.1	6.6	B

**Table 4:** Maximum velocity in mm/s during bending portion of task cycle in medial/lateral and anterior/posterior directions compared between assistance conditions of with exoskeleton (EXO) and without exoskeleton (No EXO). (note: statistically significantly different values are shown by ‘*’ symbol in the ‘Diff’ column).

	Anterior/Posterior			Medial/Lateral		
Assistance	Mean	SD	Diff (EXO v No EXO)	Mean	SD	Diff (EXO v No EXO)
EXO	356.70	86.99	*	130.00	105.55	-
No EXO	409.80	111.06		140.40	115.01	

**Table 5:** Maximum angular acceleration of the trunk in sagittal plane categorized according to assistance as exoskeleton (EXO) and without exoskeleton (No EXO); and posture as asymmetric (A) and symmetric (S). (note: statistically significantly different values are shown by ‘*’ symbol in the ‘Diff’ column).

Assistance	Maximum Angular Acceleration in Anterior/Posterior Direction During Bending (mm/s^2^)			Maximum Angular Acceleration in Anterior/Posterior Direction During Retraction (mm/s^2^)		
	Mean	SD	Diff	Mean	SD	Diff
EXO	28.87	4.68	*	32.05	5.49	*
No EXO	33.60	4.49		36.63	3.91	

**Table 6:** Overshoot percentages for bending and retraction at start/end categorized according to assistance as exoskeleton (E) and without exoskeleton (NE); and posture as asymmetric (A) and symmetric (S). (note: statistically significantly different values are shown by ‘*’ symbol in the ‘Diff’ column).

Response Variables		Asymmetric Posture			Symmetric Posture		
		E	NE	Diff (EXO v No EXO)	E	NE	Diff (EXO v No EXO)
Percent overshoot during start of bending in vertical direction (%)	Mean	7.06	7.74	*	6.18	8.18	*
	SD	3.56	2.72		3.16	3.38	
Percent overshoot during end of bending in vertical direction (%)	Mean	2.94	2.86	*	2.73	3.05	*
	SD	2.73	2.46		2.42	2.75	
Percent overshoot during end of retraction in frontal direction (%)	Mean	8.06	6.36	*	5.57	5.35	*
	SD	5.45	3.97		3.80	3.16	

**Table 7:** Outcomes of measures of stability categorized according to assistance (EXO: exoskeleton, No EXO: no exoskeleton). (note: statistically significantly different values are shown by ‘*’ symbol in the ‘Diff’ column).

Response Variable/Assistance	EXO		No EXO		Diff (EXO v No EXO)
	Mean	SD	Mean	SD	
Maximum deviationin COP direction during sustained bending (mm)	20.80	8.98	25.34	7.75	*
Mean velocity in COP during sustained bending (mm/s)	5.04	5.32	9.14	5.68	*
Sample Entropy in COP in anterior–posterior direction during sustained bending (units)	0.08	0.11	0.14	0.12	*
Sample Entropy in COP in medio-lateral direction during sustained bending (units)	0.28	0.24	0.46	0.23	*
Variance in COP in anterior–posterior direction during sustained bending (mm^2^)	39.75	48.63	25.87	21.82	*
Variance in COP in medio-lateral direction during sustained bending (mm^2^)	30.87	32.21	47.23	34.04	*
Difference in GRF beween left and right foot during sustained bending (N)	19.22	15.95	23.13	18.00	*
GRF at left foot (N)	456.05	99.79	411.96	160.12	*
GRF at right foot (N)	288.92	91.68	259.84	102.31	*

**Table 8:** Outcomes of measures of stability categorized according to posture (Asymmetry, Symmetry). (note: statistically significantly different values are shown by ‘*’ symbol in the ‘Diff’ column).

Response Variable/Posture	Asymmetric		Symmetric		
	Mean	SD	Mean	SD	Diff (Asymmetric v Symmetric)
Maximum deviationin COP direction during sustained bending (mm)	24.04	9.20	21.21	8.15	*
Mean velocity in COP during sustained bending (mm/s)	7.05	6.08	6.30	5.54	*
Variance in COP in medio-lateral direction during sustained bending (mm^2^)	45.91	37.52	29.26	27.71	*
Difference in GRF beween left and right foot during sustained bending (N)	35.62	9.17	6.39	7.71	*
GRF at left foot (N)	535.38	79.36	344.60	93.46	*
GRF at right foot (N)	202.03	75.72	350.38	46.32	*

**Table 9:** Variance (mean and SD) of COP location in mm^2^ during sustained bending in medial-lateral direction categorized based on assistance and fatigue levels in both back and legs. (note: significance is shown by uncommon letters).

Posture	Assistance	Fatigue		Mean	SD	Significance
Asymmetric	EXO	Back Fatigue Categorization	Low	31.64	35.04	B,C
			Medium	41.76	38.04	A,B
	No EXO		Low	58.06	33.76	A
			Medium	59.74	35.78	A
Symmetric	EXO		Low	23.66	24.85	C
			Medium	27.64	27.81	B,C
	No EXO		Low	33.49	31.44	B
			Medium	36.44	26.95	B,C
Asymmetric	EXO	Leg Fatigue Categorization	Low	32.39	35.21	B
			Medium	40.44	37.95	A,B
	No EXO		Low	62.22	39.59	A
			Medium	57.25	32.05	A
Symmetric	EXO		Low	23.72	24.12	C
			Medium	26.46	27.36	B,C
	No EXO		Low	28.13	29.55	B,C
			Medium	38.67	28.09	B

## Data Availability

The raw data supporting the conclusions of this article will be made available by the corresponding authors upon reasonable request.

## Appendix A

Summary of statistical comparisons between different experimental conditions for each of the calculated measures has been depicted in Table A1, Table A2, Table A3, Table A4 and Table A5. The outcomes of the statistically significant outcomes have been described in depth in subsequent sections.

**Table A1:** Measured parameters and their description.

Response Variable (Unit)	Description
B_duration (s)	Duration of bending movement in seconds.
R_duration (s)	Duration of retraction movement in seconds.
Max_Vel(x/y/z)_UB1_SUS (mm/s)	Maximum velocity of upper-back marker 1 in x: forward/backward, y: sideways, z: up/down directions, respectively.
UB1_PercentOvershoot_B_(start/end)_Z (%)	Movement of upper-back marker 1 beyond intended position before starting/after ending the bending movement in z direction in percentage.
UB1_PercentOvershoot_R_end_X (%)	Movement of upper-back marker 1 beyond intended position after completing the retraction movement in x direction in percentage.
Dist_UB1_COP_(x/y)_SUS (mm)	Distance between the upper-back marker 1 and the center of pressure in x: forward/backward, y: sideways directions during the sustained bending activity.
Max_Vel(x/y/z)_UB1_(B/R) (mm/s)	Maximum velocity of upper-back marker 1 in x: forward/backward, y: sideways, z: up/down directions.
MaxDevCOP_(SUS/SS/SE) (mm)	Maximum deviation of the center of pressure during SUS: sustained, SS: standing at start, SE: standing at end portions of task cycle.
MeanVelCOP_(SUS/SS/SE) (mm/s)	Mean velocity of the center of pressure in x: forward/backward, y: sideways during SUS: sustained, SS: standing at start, SE: standing at end portions of task cycle.
SampEn_COP(x/y)_SUS (units)	Sample entropy of the center of pressure in x: forward/backward, y: sideways during sustained portion of task cycle.
VARCOP(x/y)_SUS (mm^2^)	Variance of the center of pressure in x: forward/backward, y: sideways during sustained portion of task cycle.
FP_Difference_SUS (N)	Difference between the ground reaction force between left and right force plate during sustained portion of task cycle.
Mean_FP_(Left/Right) Forces_SUS (N)	Mean of ground reaction forces in the left/right force plate during the sustained portion of task cycle.

**Table A2:** Summary statistics showing the main and interaction effects of assist (AST), posture (P) on measures of body movement (shown effect sizes in terms of partial eta-squared (ηp^2^) are interpreted as: ηp^2^ = 0.01 indicates a small effect, ηp^2^ = 0.06 indicates a medium effect, ηp^2^ = 0.14 indicates large effect). (note: values in bold are statistically significant)

Measure	Effect	*p*-Values	ηp^2^	Measure	Effect	*p*-Values	ηp^2^
Bending duration (s)	AST	**0**	0.051	R_duration (s)	AST	**0.000**	0.114
	P	**0.001**	0.014		P	0.052	0.003
	P*AST	0.573	0.001		P*AST	0.219	0.001
Distance between upper-back and COP	AST	**0.000**	0.018	Dist_UB1_COP_y_SUS	AST	**0.000**	0.008
	P	**0.000**	0.327		P	**0.000**	0.810
	P*AST	**0.000**	0.018		P*AST	0.208	0.001
Max_Velx_UB1_B	AST	**0.000**	0.073	Max_Vely_UB1_B	AST	0.437	0.001
	P	**0.000**	0.082		P	**0.000**	0.759
	P*AST	0.157	0.001		P*AST	0.707	0.001
Max_Velx_UB1_R	AST	**0.000**	0.171	Max_Vely_UB1_R	AST	0.346	0.001
	P	**0.000**	0.064		P	**0.000**	0.824
	P*AST	**0.015**	0.005		P*AST	0.154	0.001
Max_Velx_UB1_SUS	AST	**0.002**	0.013	Max_Velz_UB1_R	AST	**0.000**	0.020
	P	0.063	0.004		P	0.147	0.002
	P*AST	0.792	0.001		P*AST	**0.005**	0.008
UB1_PercentOvershoot_B_end_Z	AST	**0.000**	0.001	UB1_PercentOvershoot_R_end_X	AST	**0.003**	0.011
	P	0.147	0.001		P	**0.000**	0.038
	P*AST	**0.005**	0.002		P*AST	**0.024**	0.006
UB1_PercentOvershoot_B_start_Z	AST	**0.000**	0.038	B_MaxAngAccxJnt_UB_Hip	AST	**0.000**	0.178
	P	0.373	0.001		P	**0.000**	0.036
	P*AST	**0.006**	0.009		P*AST	0.058	0.002
R_MaxAngAccxJnt_UB_Hip	AST	**0.000**	0.178				
	P	**0.04**	0.036				
	P*AST	**0.000**	0.002				

**Table A3:** Summary statistics showing the main and interaction effects of assist (AST), posture (P) on measures of body movement categorized according to back (FB) and leg fatigue (FL) of low (L) and medium (M) fatigue. (note: values in bold are statistically significant)

		Back Categorization		Leg Categorization	
		*p*-Values		*p*-Values	
Measure	Effect	L	M	L	M
B_duration (s)	AST	**0.000**	**0.000**	**0.000**	**0.000**
	P	**0.009**	**0.032**	0.214	**0.002**
	P*AST	0.955	0.534	0.924	0.380
Dist_UB1_COP_x_SUS	AST	**0.000**	0.141	**0.000**	**0.031**
	P	**0.000**	**0.000**	**0.000**	**0.000**
	P*AST	**0.000**	**0.006**	**0.000**	**0.005**
Dist_UB1_COP_y_SUS	AST	**0.001**	**0.000**	0.792	**0.000**
	P	**0.000**	**0.000**	**0.000**	**0.000**
	P*AST	0.594	0.151	0.349	**0.000**
Max_Velx_UB1_B	AST	**0.000**	**0.000**	**0.000**	**0.000**
	P	**0.000**	**0.000**	**0.000**	**0.000**
	P*AST	0.196	0.530	0.720	0.247
Max_Velx_UB1_R	AST	**0.000**	**0.000**	**0.000**	**0.000**
	P	**0.000**	**0.000**	**0.000**	**0.000**
	P*AST	0.092	0.143	0.173	0.077
Max_Velx_UB1_SUS	AST	**0.003**	0.111	**0.000**	0.153
	P	0.069	0.320	0.762	**0.024**
	P*AST	0.743	0.396	0.480	0.904
Max_Vely_UB1_B	AST	0.744	0.441	0.202	0.573
	P	**0.000**	**0.000**	**0.000**	**0.000**
	P*AST	0.693	0.723	0.192	0.287
Max_Vely_UB1_R	AST	0.955	0.243	**0.014**	0.164
	P	**0.000**	**0.000**	**0.000**	**0.000**
	P*AST	0.206	0.356	**0.007**	0.348
Max_Velz_UB1_B	AST	0.051	0.684	**0.049**	0.520
	P	0.130	0.275	**0.021**	**0.049**
	P*AST	0.369	0.744	0.302	0.700
Max_Velz_UB1_R	AST	**0.000**	0.163	**0.002**	0.055
	P	0.541	**0.011**	0.273	**0.001**
	P*AST	**0.022**	0.149	**0.011**	0.320
R_duration (s)	AST	**0.000**	**0.000**	**0.000**	**0.000**
	P	0.900	**0.014**	**0.034**	0.453
	P*AST	0.314	0.636	0.433	0.259
UB1_PercentOvershoot_B_end_Z	AST	0.766	0.387	0.841	0.599
	P	0.286	0.261	0.156	0.255
	P*AST	0.387	0.418	0.626	0.085
UB1_PercentOvershoot_B_start_Z	AST	**0.007**	**0.000**	0.171	**0.013**
	P	0.051	0.425	**0.001**	**0.041**
	P*AST	**0.028**	0.139	**0.025**	0.332
UB1_PercentOvershoot_R_end_X	AST	**0.002**	0.285	**0.000**	0.895
	P	**0.001**	**0.000**	**0.004**	**0.000**
	P*AST	0.106	0.149	0.091	0.438
B_MaxAngAccxJnt_UB_Hip	AST	**0.000**	**0.000**	**0.000**	**0.000**
	P	**0.000**	0.167	**0.000**	**0.000**
	P*AST	0.052	0.715	**0.040**	0.808
R_MaxAngAccxJnt_UB_Hip	AST	**0.000**	**0.000**	**0.000**	**0.000**
	P	0.22	0.119	**0.025**	0.773
	P*AST	**0.000**	**0.000**	**0.000**	**0.000**

**Table A4:** Summary statistics showing the main and interaction effects of assist (AST), posture (P) on measures of stability (shown effect sizes in terms of partial eta-squared (ηp^2^) are interpreted as: ηp^2^ = 0.01 indicates a small effect, ηp^2^ = 0.06 indicates a medium effect, ηp^2^ = 0.14 indicates large effect). (note: values in bold are statistically significant)

**Sustained Bending Measure**	**Effect**	***p*-Values**	**ηp^2^**	**Measure**	**Effect**	***p*-Values**	**ηp^2^**
FP_Difference_SUS	AST	**0.000**	0.007	SampEn_COPx_SUS	AST	**0.000**	0.065
	P	**0.000**	0.719		P	**0.006**	0.008
	P*AST	0.350	0.001		P*AST	0.515	0.001
MaxDevCOP_SUS	AST	**0.000**	0.061	SampEn_COPy_SUS	AST	**0.000**	0.121
	P	**0.000**	0.028		P	0.104	0.003
	P*AST	**0.010**	0.008		P*AST	**0.002**	0.011
Mean_FP_LeftForces_SUS	AST	**0.000**	0.035	VARCOPx_SUS	AST	**0.000**	0.028
	P	**0.000**	0.556		P	0.363	0.001
	P*AST	**0.000**	0.006		P*AST	0.608	0.001
Mean_FP_RightForces_SUS	AST	**0.000**	0.015	VARCOPy_SUS	AST	**0.000**	0.052
	P	**0.000**	0.558		P	**0.000**	0.063
	P*AST	0.774	0.001		P*AST	**0.011**	0.008
MeanVelCOP_SUS	AST	**0.000**	0.117				
	P	**0.028**	0.006				
	P*AST	**0.000**	0.016				
**Static Standing at Start Measure**	**Effect**	***p*-Values**	**ηp^2^**	**Measure**	**Effect**	***p*-Values**	**ηp^2^**
MaxDevCOP_SS	AST	**0.000**	0.029	MeanVelCOP_SS	AST	**0.000**	0.113
	P	0.653	0.001		P	0.333	0.001
	P*AST	0.811	0.001		P*AST	**0.001**	0.014
**Static Standing at End Measure**	**Effect**	***p*-Values**		**Measure**	**Effect**	***p*-Values**	
MaxDevCOP_SE	AST	**0.000**	0.028	MeanVelCOP_SE	AST	**0.000**	0.115
	P	**0.001**	0.014		P	0.315	0.001
	P*AST	0.997	0.001		P*AST	**0.001**	0.013

**Table A5:** Summary statistics showing the main and interaction effects of assist (A), posture (P) on measures of stability categorized according to back (FB) and leg fatigue (FL). (note: values in bold are statistically significant).

		*p*-Values (FB)		*p*-Values (FL)	
Measure	Effect	L	M	L	M
Sustained Bending					
FP_Difference_SUS	AST	**0.008**	**0.000**	**0.005**	**0.000**
	P	**0.000**	**0.000**	**0.000**	**0.000**
	P*AST	0.110	0.917	**0.015**	0.346
MaxDevCOP_SUS	AST	**0.000**	**0.000**	**0.000**	**0.000**
	P	**0.016**	**0.000**	**0.031**	**0.000**
	P*AST	**0.031**	0.108	**0.011**	0.303
Mean_FP_LeftForces_SUS	AST	**0.000**	**0.000**	**0.000**	**0.000**
	P	**0.000**	**0.000**	**0.000**	**0.000**
	P*AST	**0.007**	**0.025**	**0.031**	**0.005**
Mean_FP_RightForces_SUS	AST	**0.000**	**0.001**	**0.000**	**0.001**
	P	**0.000**	**0.000**	**0.000**	**0.000**
	P*AST	0.773	0.824	0.483	0.368
MeanVelCOP_SUS	AST	**0.000**	**0.000**	**0.000**	**0.000**
	P	0.354	**0.030**	0.274	**0.034**
	P*AST	**0.005**	**0.007**	**0.000**	0.109
SampEn_COPx_SUS	AST	**0.000**	**0.000**	**0.000**	**0.000**
	P	0.060	**0.040**	0.132	**0.023**
	P*AST	0.919	0.290	0.495	0.857
SampEn_COPy_SUS	AST	**0.000**	**0.000**	**0.000**	**0.000**
	P	0.617	0.072	0.471	0.104
	P*AST	**0.018**	**0.026**	**0.000**	0.344
VARCOPx_SUS	AST	**0.000**	**0.008**	**0.000**	**0.008**
	P	0.603	0.338	0.392	0.814
	P*AST	0.783	0.513	0.725	0.646
VARCOPy_SUS	AST	**0.000**	**0.000**	**0.000**	**0.000**
	P	**0.000**	**0.000**	**0.000**	**0.000**
	P*AST	**0.008**	0.235	**0.002**	0.470
Static Standing at Start					
MaxDevCOP_SS	AST	**0.000**	**0.005**	0.594	**0.000**
	P	0.113	**0.032**	0.678	0.821
	P*AST	0.473	0.272	0.927	0.907
MeanVelCOP_SS	AST	**0.000**	**0.000**	**0.000**	**0.000**
	P	0.929	0.207	0.689	0.271
	P*AST	**0.011**	**0.013**	**0.000**	0.174
Static Standing at End					
MaxDevCOP_SE	AST	**0.000**	**0.005**	0.680	**0.000**
	P	0.263	**0.001**	**0.006**	0.180
	P*AST	0.333	0.376	0.598	0.265
MeanVelCOP_SE	AST	**0.000**	**0.000**	**0.000**	**0.000**
	P	0.913	0.198	0.680	0.263
	P*AST	**0.014**	**0.013**	**0.000**	0.176
